# Intraocular Pressure in Clinically Normal Alpacas: A Systematic Review and Meta-Analysis of Tono-Pen and TonoVet Tonometry

**DOI:** 10.3390/ani16203259

**Published:** 2026-10-16

**Authors:** Yu-Ting Hsiao, Pei-Chuan Hsu, Jyh-Mirn Lai

**Affiliations:** 1 Farm Animal Research Group, Department of Veterinary Medicine, National Chiayi University, No. 580, XinMin Rd., Chiayi City 60004, Taiwan; syt1102@gmail.com; 2 Ph. D. Program of Agricultural Science, No.300 Syuefu Rd., Chiayi City 600048, Taiwan

**Keywords:** alpaca, *Vicugna pacos*, intraocular pressure, applanation, Tono-Pen, rebound, TonoVet, tonometry, meta-analysis, veterinary ophthalmology, reference values

## Abstract

Intraocular pressure (IOP) is a critical physiological indicator of ocular health in canines, felines, and equines, yet a precise, tool-independent baseline remains inconsistent in South American camelids. This study quantitatively evaluated the comparability and potential bias between Tono-Pen applanation and TonoVet rebound tonometry in clinically normal alpacas (*Vicugna pacos*). Following a systematic review of the literature published between 1990 and 2026 across MEDLINE, Web of Science, and CAB abstracts, data from six eligible studies encompassing 320 healthy eyes were extracted, including 500 tests. Tono-Pen pooled mean: 15.84 mmHg (95% CI: 14.06–17.62) and TonoVet pooled mean: 15.89 mmHg (95% CI: 13.79–17.99), respectively. Paired comparison demonstrated a clinically negligible pooled mean difference of +0.58 mmHg (95% CI: −1.85 to +3.02) between devices. Subgroup and meta-regression analyses confirmed that tonometer type did not significantly influence IOP measurements (*β* = −0.36 mmHg; 95% CI: −4.86 to +4.17; *p* = 0.85), though notable heterogeneity existed (tau^2^ ≒ 1.74–2.60; *I*^2^ > 90%) due to variations in populations and operator techniques. In conclusion, both Tono-Pen and TonoVet yield comparable intraocular pressure measurements. These findings provide an evidence-based pooled estimate of normal intraocular pressure in healthy alpacas, which is crucial for diagnosing ophthalmic pathologies like glaucoma and uveitis in camelid veterinary medicine.

## Simple Summary

Checking the pressure inside the eye is an important step in alpaca health care, since this measurement can detect problems like glaucoma early. Veterinarians usually rely on two handheld instruments: one that touches the eye gently after numbing drops, and another that uses a light probe without anesthesia. Until now, it was uncertain whether these tools provide equally reliable results in alpacas. By combining information from several previous studies, our analysis demonstrated that the average difference in readings between the two devices was small and not statistically significant, indicating broadly comparable performance. These findings provide preliminary evidence that either instrument is appropriate for routine eye examinations in alpacas, provided that veterinarians use the same device consistently for individual patients and follow standardized protocols. This work helps fill an important knowledge gap and supports more informed decision-making in long-term vision care for alpacas.

## 1. Introduction

Intraocular pressure (IOP) is a fundamental physiological parameter that reflects the dynamic balance between aqueous humor production and outflow and serves as a critical indicator for detecting glaucoma, uveitis, and other intraocular pathologies in both humans and animals [1]. Establishing reliable species-specific reference values remains challenging because tonometric readings are mediated by biological and methodological factors, including corneal biomechanics [2], animal handling, environmental conditions, examiner experience, and the tonometry technique employed [3]. Veterinary practice has largely transitioned to two portable instruments: applanation tonometry (Tono-Pen) and rebound tonometry (TonoVet). However, discrepancies between these devices are documented across diverse domestic species [3,4].

These discrepancies stem from fundamentally different measurement principles. The Tono-Pen applanates a flat central corneal area and requires topical anesthesia, rendering it sensitive to patient reactions, probe contact angle, and central corneal thickness (CCT) [3,5]. In contrast, the TonoVet uses a low-mass electromagnetic probe that briefly impacts and rebounds off the cornea without topical anesthesia, offering superior feasibility in field settings and uncooperative patients. Reports note that readings may vary by device, timing, corneal properties, and corneal curvature and stromal rigidity, which affect readings differently in humans [2,5] and animals [6,7].

These biomechanical interactions are particularly pronounced in alpacas, which possess large, horizontally oval corneas with variable stromal thickness and marked curvature [5,8]. In clinically healthy alpacas, reported normative IOP spans 12 to 19 mmHg [1,3,6,9,10,11], but values vary substantially across modalities. Studies using applanation tonometry (Tono-Pen) report mean values of 14.85 ± 2.70 mmHg and 16.14 ± 3.74 mmHg [1,5], with some studies approaching 19 mmHg [9]. Rebound tonometry (TonoVet) yields readings ranging from 12.51 to 17.20 mmHg [2,3,6,9]. Previous comparative studies yielded conflicting direction offsets [3,9], which often diverge further at elevated intraocular pressures. Because existing camelid investigations rely on small cohort sizes and heterogeneous protocols, an objective consensus regarding normative intervals and device interchangeability remains absent.

To resolve these discrepancies, the present study conducted a systematic review and meta-analysis to: (1) establish a robust pooled normative estimate of mean IOP in clinically healthy alpacas, and (2) formally evaluate the agreement and statistical equivalence between Tono-Pen and TonoVet measurements. By clarifying systematic bias and instrument comparability, this work provides an evidence-based clinical reference for camelid ophthalmology and guides protocol standardization in veterinary practice.

## 2. Materials and Methods

The systematic review and meta-analysis were planned, conducted, and reported in strict accordance with the Preferred Reporting Items for Systematic Reviews and Meta-Analyses (PRISMA 2020) guidelines [12].

### 2.1. Search Strategy and Study Selection

A comprehensive literature search was conducted on 30 March 2026, to identify published reports evaluating intraocular pressure (IOP) in camelids using either Tono-Pen applanation tonometry or TonoVet rebound tonometry. Systematic searches were performed across MEDLINE/PubMed, Web of Science, and CAB Abstracts, supplemented by secondary searches of Google Scholar and ProQuest Dissertations. No language or geographical restrictions were applied. The search strategy combined controlled vocabulary (e.g., MeSH terms) and free-text keywords, including “llama”, “alpaca”, and “tonometry”.

**Figure 1:**
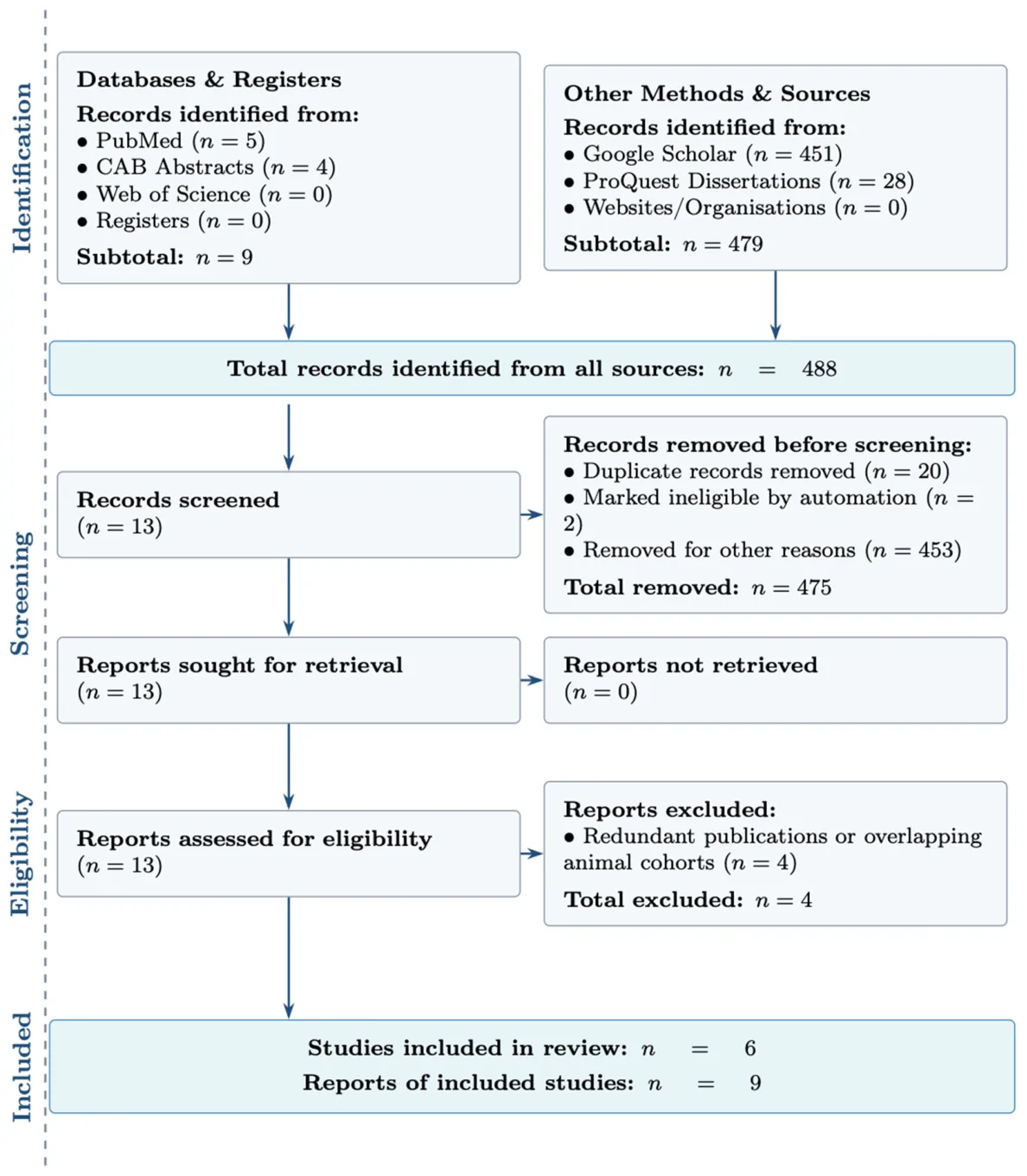
PRISMA 2020 flow diagram detailing the study selection process. A total of 488 records are identified from databases (*n* = 9) and other methods (*n* = 479). After removing duplicates (*n* = 20), automated exclusions (*n* = 2), and other ineligible records (*n* = 453), 13 reports are screened and assessed for eligibility. Four reports are excluded due to redundant publications or overlapping cohorts, resulting in a final inclusion of 6 studies yielding 9 independent datasets for quantitative synthesis.

The overall screening and identification process followed the Preferred Reporting Items for Systematic Reviews and Meta-Analyses (PRISMA 2020) statement [12], and Figure 1 depicts the corresponding selection workflow. The protocol for this systematic review was not prospectively registered in an international registry (such as PROSPERO or the Open Science Framework) because PROSPERO prioritizes human health outcomes and typically excludes animal physiological studies lacking direct human health relevance; however, all eligibility criteria, screening workflows, and analytical pipelines were planned a priori and strictly adhered to throughout the review process.

To minimize selection bias and ensure methodological rigor, three reviewers (Y.-T.H., P.-C.H., and J.M.L.) performed the study selection process independently. In the first stage, two reviewers (Y.-T.H. and P.-C.H.) independently screened the titles and abstracts of all retrieved records against the predefined eligibility criteria. We manually identified and removed duplicate records across databases. In the second stage, the same two reviewers independently evaluated the full text of the remaining potentially eligible reports. Throughout both stages, any disagreements or uncertainties regarding study inclusion were resolved through comprehensive discussion or, when necessary, via adjudication by a third senior reviewer (J.M.L.).

Studies were included if they met the following criteria: (1) reported mean IOP with standard deviation (SD) or provided sufficient raw data to calculate these parameters; (2) specified the number of eyes examined; (3) involved clinically healthy subjects without evidence of ocular disease; and (4) were prospective or retrospective observational studies, cross-sectional cohort surveys or diagnostic instrument comparison studies.

Studies were excluded if they lacked quantitative IOP data, utilized experimental models of ocular disease, were non-camelid species without extractable alpaca subgroups, or were published only as abstracts, reviews, or editorials without accessible empirical data.

### 2.2. Data Extraction and Analysis Methods

For each eligible study, we recorded the following variables in a standardized spreadsheet: first author, publication year, number of eyes examined (*N*), mean IOP (mmHg), standard deviation (SD), and measurement technique (Tono-Pen or TonoVet).

For studies reporting both tonometry methods, the mean and SD for each device were extracted separately. To avoid double-counting and unit-of-analysis error, for primary pooled normative reference calculations, animal subjects evaluated with both devices within the same study [3,10] were treated as paired observations. For device comparative evaluations, within-subject paired subsets (*N* = 80 in [10]; *N* = 50 in [3]) were isolated specifically for head-to-head bias and equivalence analysis.

### 2.3. Risk of Bias Assessment

The methodological quality of the included studies was independently assessed using the Joanna Briggs Institute (JBI) Critical Appraisal Checklist for Analytical Cross-Sectional Studies [13,14]. The checklist evaluates eight domains: (Q1) clarity of inclusion criteria; (Q2) description of subjects and study setting; (Q3) validity and reliability of measurement of exposure; (Q4) objective and standardized criteria; (Q5) identification of confounders; (Q6) strategies to control confounders; (Q7) validity of outcome measurement; and (Q8) appropriateness of statistical analysis. These included participant selection, measurement validity and reliability, identification and management of confounding factors, and appropriateness of statistical analyses. Each item was rated as “Yes”, “No”, or “Unclear”. Overall risk of bias was categorized as low (≥6 Y), moderate (4–5 Y), or high (≤3 Y).

### 2.4. Statistical Analysis

Data cleaning, extraction, and transformation were performed in Python (version 3.14) and R (version 4.5.1). Some libraries, such as ‘pandas’ and ‘numpy’, were used if necessary. DerSimonian–Laird random-effects model was used to account for anticipated between-study heterogeneity:

Subgroup Meta-analysis of Baseline IOP by Modality (Tono-Pen and TonoVet separately):

A single-arm meta-analysis was performed separately to estimate the device-specific pooled mean IOP values and 95% confidence intervals (95% CIs). Study weights were computed via the inverse-variance method.

$$\omega_{i}=\frac{1}{{\mathrm{SE}}^{2}+\tau^{2}}$$

where ${\mathrm{SE}}_{i}=\frac{{\mathrm{SD}}_{i}}{\sqrt{N_{i}}}$ and $\tau^{2}$ denotes the between-study variance.

For studies that utilized both devices within identical subjects under standardized conditions, the mean difference was defined as:

(MD = Mean_TonoVet_ − Mean_Tono-Pen_)

Because primary papers did not report the standard deviation of paired differences (SD_diff_) or intra-eye correlation coefficients (*r*), treating paired cohorts as independent groups artificially inflates variance. Therefore, within-study variance of MD was modeled under explicit paired repeated-measures covariance:


$$\mathrm{Var}(\mathrm{MD})=\frac{{\mathrm{SD}}_{\mathrm{TonoPen}}^{2}+{\mathrm{SD}}_{\mathrm{TonoVet}}^{2}-2r*{\mathrm{SD}}_{\mathrm{TonoPen}}*{\mathrm{SD}}_{\mathrm{TonoVet}}}{N}$$

A sensitivity analysis across biologically plausible correlation boundaries (*r* ∈ [0.5, 0.8]) was conducted to ensure robust pooled inference.

### 2.5. Heterogeneity and Meta-Regression Analyses

Between-study statistical heterogeneity was evaluated using Cochran’s *Q*-test (with significance threshold set at *p* < 0.10) and quantified using the Higgins *I*^2^ index and between-study variance (*τ*^2^). *I*^2^ values of approximately 25%, 50%, and 75% were interpreted as low, moderate, and high heterogeneity, respectively. To determine whether tonometer modality systematically accounted for between-study variance, a weighted random-effects meta-regression was conducted:

$$IOP_{i}=\beta_{0}+\beta_{1}(Method_{i})+\varepsilon_{i}$$

where Method_i_ was parameterized as an indicator covariance (0 = Tono-Pen, 1 = TonoVet), statistical significance was defined as *p* < 0.05.

### 2.6. Clinical Equivalence Testing (TOST Framework)

Conventional null-hypothesis significance testing is structurally incapable of confirming equivalence (as *p* > 0.05 merely reflects the absence of evidence for difference, rather than evidence of comparability). Therefore, device equivalence was evaluated using the two one-sided tests (TOST) procedure at a significance level of *ɑ* = 0.05, corresponding to a two-sided 90% confidence interval.

The null hypothesis postulated that the true mean difference between devices exceeded predefined clinically acceptable margins ([−θ, +θ]):


$$H_{01}:\Delta \leq -\theta_{L}\mathrm{vs}H_{11}:\Delta \geq +\theta_{L}$$

$$H_{02}:\Delta \geq +\theta_{U}\mathrm{vs}H_{12}:\Delta <+\theta_{U}$$

Statistical equivalence was declared if both one-sided null hypotheses were rejected (*p*_TOST_ = max (*p*_1_,*p*_2_) < 0.05), demonstrating that the 90% CI for the mean difference fell entirely within the equivalence bounds. Because a universal clinical margin has not been established in camelids, four prospective clinical margins were systematically assessed: ±1.0, ±1.5, ±2.0, and ±2.5 mmHg. TOST evaluations were performed across the paired-correlation corridor (r = 0.5, 0.6, 0.7, 0.8) to account for paired-covariance uncertainty.

## 3. Results

### 3.1. Study Selection and Quantity of Research Available (PRISMA Flow)

The PRISMA 2020 flow diagram (Figure 1) summarizes the study selection process and identification results. A total of 488 records were initially identified, comprising 9 records retrieved from primary database searches (MEDLINE, Web of Science, and CAB Abstracts) and 479 records through other sources, including Google Scholar (*N* = 451) and ProQuest Dissertations (*N* = 28). Before screening, 475 records were removed, including duplicates (*N* = 20), records marked ineligible by automation (*N* = 2), and records removed for other reasons (*N* = 453). The other reasons include ineligible document types, such as books, citation-only entries, or editorial materials without primary empirical data; irrelevant scope, such as non-veterinary or non-target species or unrelated disciplines; and language limitations, such as non-English publications with full text unavailable. We screened the remaining 13 records by title and abstract, and sought and retrieved all 13 reports for full-text assessment. After full-text evaluation, four reports were excluded due to redundant publications or overlapping animal cohorts (*N* = 4). Ultimately, we included six studies, comprising nine independent datasets, in the final quantitative synthesis. In total, the meta-analysis included 320 eyes from clinically healthy alpacas (*Vicugna pacos*). According to the Faulkner report (2020), 92 individual eyes (out of 320) were tested [3]. The 92 eyes were measured using TonoVet. Again, 50 of the 92 eyes were measured with the TonoVet and Tono-Pen after local anesthesia. Thus, 100 tests were performed [3]. According to the original data, 80 eyes underwent two measurements [10]. Thus, 500 tests (320 + 180) were included. All included studies were conducted between 1990 and 2026 across various clinical and field settings, with minor methodological variations in animal restraint, anesthesia use, and measurement timing. Sample sizes ranged from 20 to 92 eyes per study, with reported mean intraocular pressure (IOP) values spanning 12.51 to 19.00 mmHg. All studies used either applanation tonometry Tono-Penn) or rebound tonometry (TonoVet) to measure IOP in clinically normal eyes using non-invasive methods. Table 1 summarizes the extracted data, including mean IOP, standard deviation (SD), and tonometer models.

### 3.2. Risk of Bias Findings

The methodological quality of the included studies was appraised using the Joanna Briggs Institute (JBI) Critical Appraisal Checklist for Analytical Cross-Sectional Studies (Table 2).

### 3.3. Device-Specific Pooled Normative IOP (Single-Arm Meta-Analysis)

**Figure 2:**
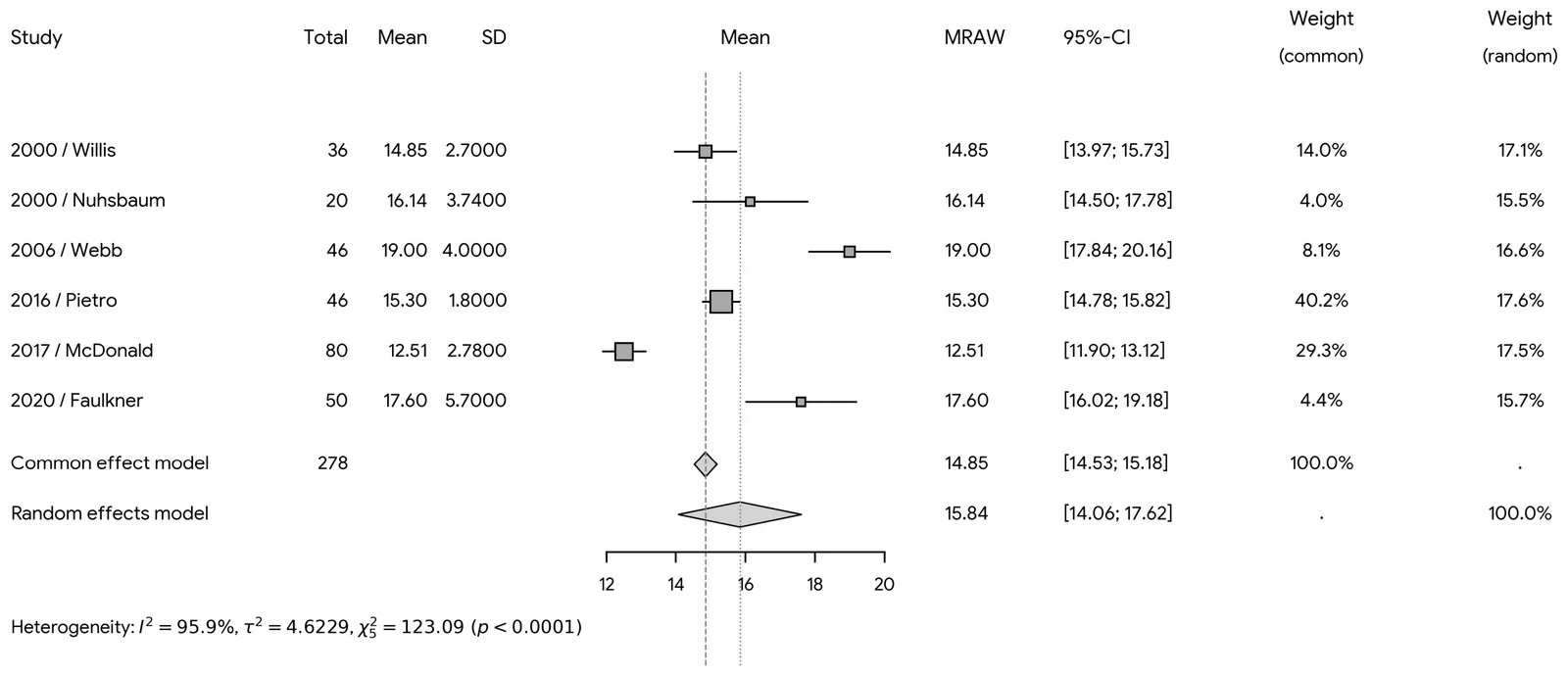
Forest plot of mean intraocular pressure (IOP) measured using the Tono-Pen tonometer. Each horizontal line represents an individual study’s mean ± 95% confidence interval. The diamond at the bottom indicates the pooled estimate derived from a DerSimonian–Laird random-effects model. Considerable heterogeneity is observed (τ^2^ = 4.62), but the pooled IOP remains near 16 mmHg across studies [1,3,6,9,10,11].

Single-arm meta-analyses using the DerSimonian–Laird random-effects model were conducted separately for each tonometric modality:

Synthesizing the six datasets utilizing Tono-Pen (n = 278 eyes; 1,4, 6, 8, 7, 2) yielded a pooled mean IOP of 15.84 mmHg (95% CI: 14.06–17.62 mmHg) under the random-effects model (fixed-effect mean: 14.85 mmHg, 95% CI: 14.53–15.18 mmHg; Figure 2). Substantial between-study heterogeneity was observed (*Q* = 123.09, *df* = 5, *p* < 0.001; *τ*^2^ = 4.62; *I*^2^ = 95.9%).

**Figure 3:**
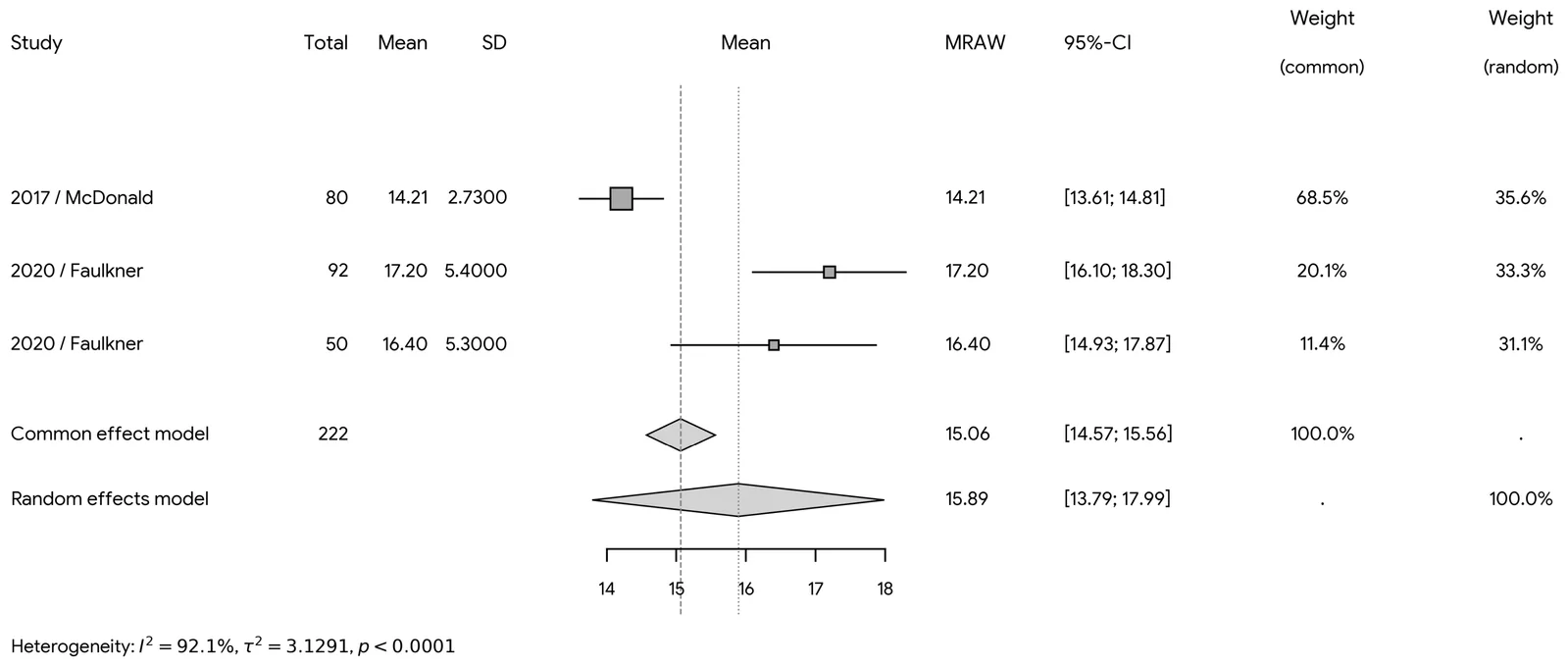
Forest plot of the single-method meta-analysis for intraocular pressure (IOP) measured via TonoVet rebound tonometry. Each horizontal line represents the mean IOP and 95% confidence interval (CI) of an individual dataset. The gray squares indicate the weight assigned to each study under the common effect model. The diamonds at the bottom represent the pooled estimates derived from the common effect model (top diamond, 15.06 mmHg) and the DerSimonian–Laird random-effects model (bottom diamond, 15.89 mmHg), respectively. High and statistically significant between-study heterogeneity is observed (*I*^2^ = 92.1%, *τ*^2^ = 3.1291, *Q* = 25.40, *p* < 0.0001) [3,10].

Synthesizing the three datasets utilizing TonoVet (*N* = 222 measurements: [10]; *N* = 80, [3] *N* = 92, and [3] paired subset *N* = 50) yielded a random-effects pooled mean IOP of 15.89 mmHg (95% CI: 13.79–17.99 mmHg; fixed-effect mean: 15.06 mmHg, 95% CI: 14.57–15.56 mmHg; Figure 3). Heterogeneity within the rebound tonometry group was similarly elevated (*Q* = 25.40, df = 2, *p* < 0.001; *τ*^2^ = 3.13; *I*^2^ = 92.1).

Comparing the two device baselines revealed that the overall pooled normative IOP estimates are nearly identical between modalities, with an indirect between-group difference of −0.34 mmHg (95% CI: −4.86 to +4.17 mmHg; Table 3, Part A).

### 3.4. Head-to-Head Comparative Meta-Analysis and Systematic Bias

Two studies directly compared tonometry on identical subjects (Table 1). In McDonald et al. (2017) (*N* = 80), the mean IOP recorded by Tono-Pen was 12.51± 2.78 mmHg, compared to 14.21 ± 2.73 mmHg for TonoVet, representing an offset of +1.70 mmHg (TonoVet higher) [10]. In Faulkner et al. (2020) (*N* = 50), the mean IOP was 17.6 ±5.7 mmHg for Tono-Pen versus 16.4± 5.3 mmHg for TonoVet, corresponding to an offset of +1.20 mmHg (Tono-Pen higher) [3].

When evaluating the direct paired mean difference (MD = Mean_TonoVet_ − Mean_Tono-Pen_ across the comparative studies under a random-effects model incorporating paired covariance (*r* ∈ [0.5, 0.8]), the pooled mean difference was +0.58 mmHg (95% CI: −1.85 to +3.02 mmHg) (Table 3, Part B). While individual investigations showed conflicting directional offsets between devices (+1.70 mmHg favoring TonoVet [10], vs. +1.20 mmHg favoring Tono-Pen [3], the overall pooled confidence interval spanned zero, confirming that the absolute pooled discrepancy between the two instruments remains clinically negligible.

### 3.5. Clinical Equivalence Testing (TOST Sensitivity Analysis)

The results of the TOST sensitivity analysis across predefined clinical equivalence margins (±∆ = 1.0, 1.5, 2.0, 2.5 mmHg) and intra-eye correlation coefficients (*r* ∈ [0.5, 0.8]) are summarized in Table 4.

Neither study met the criteria at the narrow margins (±1.0 and ±1.5 mmHg) for statistical equivalence at any correlation level. Because the observed mean differences (+1.70 and +1.20 mmHg) fell near or exceeded these thresholds, the 90% confidence intervals shifted substantially outside the equivalence corridors.

Statistical equivalence was not achieved at the intermediate margin (±2.0 mmHg) in either cohort. In McDonald et al., the lower bound of the 90% CI remained within limits, but the upper bound reached [+2.02, +2.21] mmHg. Similarly, in Faulkner et al., wider sample variance expanded the 90% CI upper bound ([+2.03, +2.51] mmHg), narrowly crossing the +2.0 mmHg bound even under high correlation (*r* = 0.8, *p*_TOST_ = 0.056).

Both studies demonstrated statistical equivalence at the broad margin (± 2.5 mmHg). In McDonald et al., the 90% CIs ([+1.38, +2.02] mmHg at *r* = 0.8; [+1.19, +2.21] mmHg at *r* = 0.5) fell entirely within ±2.5 mmHg (*p*_TOST_ < 0.01 across all *r*). In Faulkner et al., equivalence was supported for all correlations with *r* ≥ 0.6 *(p*_TOST_ ≤ 0.034), with the 90% CI spanning [+0.19, +2.21] mmHg at *r* = 0.7.

### 3.6. Meta-Regression and Sources of Heterogeneity

Visual inspection of the effect sizes and the forest plots indicated that two studies [6,9] contributed most strongly to the observed heterogeneity, reporting higher mean IOP values and greater dispersion than other reports.

**Figure 4:**
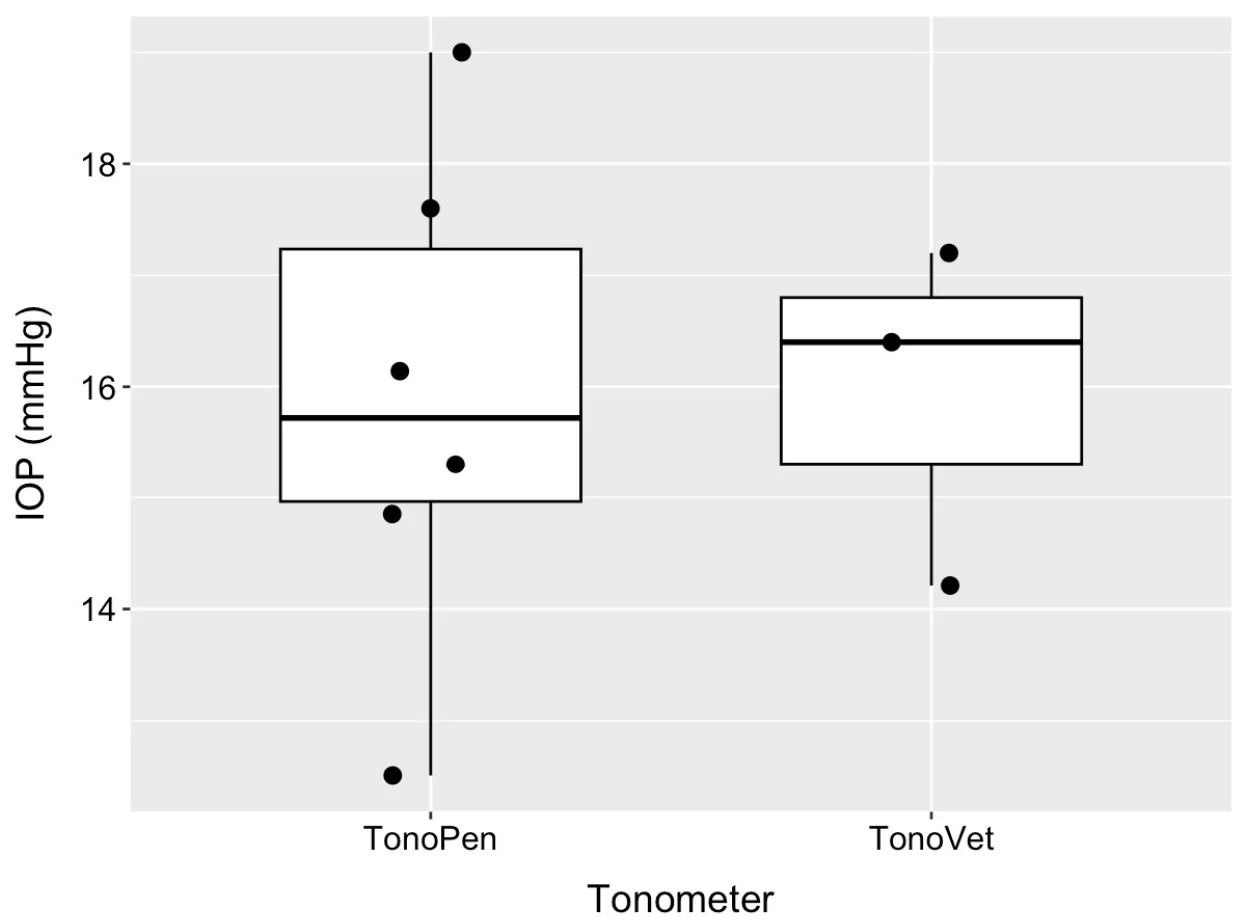
Distribution of alpaca intraocular pressure (IOP) across studies and meta-regression comparison between Tono-Pen and TonoVet tonometers. Boxplots display the median and interquartile range (IQR) of IOP values (mmHg) recorded by each device, with jittered points representing individual study/data observations. Individual black dots denote the reported mean IOP of each included dataset (k = 6 for Tono-Pen, k = 3 for TonoVet).

To assess whether tonometer modality systematically accounted for between-study variance, we conducted random-effects meta-regression. The regression model confirmed that tonometer type was not a statistically significant moderator of measured IOP (*β* = −0.36 mmHg; 95% CI: −4.86 to +4.17; *p* = 0.85) (Table 3; Figure 4). This demonstrated that device modality itself did not account for the observed between-study heterogeneity (*τ*^2^ ≒ 1.74–2.60). Instead, variations in animal restraint techniques, examiner experience, physical environment, and topical anesthetic administration account for the variability among published studies.

## 4. Discussion

To our knowledge, the present meta-analysis is the first systematic review and quantitative effort to estimate the pooled mean of intraocular pressure (IOP) in clinically healthy alpacas and to formally assess the diagnostic agreement and statistical equivalence between the two tonometry devices. The present analysis suggests the two devices are clinically interchangeable but are still not considered mathematically interchangeable.

### 4.1. Normative Pooled IOP Baseline in Healthy Alpacas

Across the studies analyzed, the random-effects pooled mean IOP in alpacas was approximately 15–16 mmHg, consistent with values reported for other small ruminants and South American camelids [11]. Single-arm syntheses yielded virtually identical baseline estimates between modalities: 15.84 mmHg (95% CI: 14.06–17.62) for Tono-Pen and 15.89 mmHg (95% CI: 13.79–17.99) for TonoVet. These pooled estimates are biologically plausible and align well with physiological intervals established in related domestic and wild ruminants, including llamas (13.2–16.8 mmHg), dromedary camels (13.0–19.0 mmHg), sheep (10.5–14.0 mmHg), goats (11.0–15.5 mmHg), and dogs (around 6.55–11.45 mmHg) [7,11].

From an evidentiary standpoint, establishing this pooled normative range of 14.0 to 18.0 mmHg provides a quantitative reference interval for veterinary clinicians. Deviations below 10 mmHg or above 20–22 mmHg should be interpreted with high clinical suspicion for ocular hypotony (secondary to uveitis or systemic dehydration) or ocular hypertension (early glaucomatous neuropathy), respectively [1].

### 4.2. Technical Discrepancies and Systematic Device Bias

Although the overall device-specific baselines appear consistent across studies, direct within-subject comparisons conducted by the authors revealed opposing directional biases between the devices [3,10]. Beyond fundamental differences in the devices’ methods of physical contact, additional factors may impact these results and warrant further investigation in future research. Factors that cause the different readings have been reported in some studies. Diurnal variation causes large differences in dogs [7] and humans [2,5,15]. Hence, it is suggested to test IOP at the same time each day. However, this factor has not been well mentioned in the six reports [1,3,6,9,10,11]. Therefore, detecting IOP at the same time every day would avoid interference and reduce the variation.

As mentioned, the Tono-Pen flattens a fixed 1.0 mm^2^ area of central cornea and requires topical anesthesia. In alpacas, topical proparacaine or tetracaine administration and the physical presence of a larger footplate frequently provoke mild patient apprehension, head shaking, and subclinical reflex blepharospasm [3]. Sustained eyelid squeezing directly compresses the globe and increases episcleral venous pressure, introducing an artifactual upward drift in IOP. Furthermore, mechanical applanation is highly sensitive to central corneal thickness (CCT) and stromal rigidity. Thus, flattening a thick, stiff camelid cornea requires higher transducer resistance, skewing readings [16].

Conversely, the TonoVet propels a low-mass, magnetized probe that briefly impacts the cornea and then bounces off without topical anesthesia. Its negligible contact duration eliminates ocular surface irritation and reflex orbicularis oculi contraction. However, rebound velocity assumes standard species-specific corneal elasticity and curvature. The horizontally oval geometry and marked stromal curvature of the alpaca cornea may subtly dampen deceleration, resulting in biased readings compared with applanation [16,17].

### 4.3. Clinical Interpretation of TOST Equivalence Corridors

Our two one-sided tests’ sensitivity analysis of the two one-sided tests provides actionable boundaries for determining whether Tono-Pen and TonoVet can be interchanged in veterinary practice, for the following reasons. Firstly, regarding narrow margins, statistical equivalence was firmly rejected across both comparative studies (*p*_TOST_ ≥ 0.27). Because the absolute systematic offset observed in individual studies (+1.20 to 1.70 mmHg in opposite directions) consumed almost the entire tolerance windows, the devices cannot be considered mathematically interchangeable under a stringent threshold. Under the intermediate margin, neither study confirmed equivalence. One report stated that the 90% CI upper bound reached +2.02 to 2.21 mmHg [10], while another report with wide sample dispersion [2] pushed the upper confidence limit to +2.03 to 2.51 mmHg (even at *r* = 0.8, *p* = 0.56). Under the broad margin condition, both studies robustly satisfied statistical equivalence across biologically plausible correlation levels (*r* ≥ 0.6, *p*_TOST_ ≤ 0.034).

For initial screening and general herd surveillance, an inter-device variance of ±2.5 mmHg is clinically inconsequential, as it falls well within the natural circadian oscillations and handling-stress fluctuations. Either device is appropriate for routine baseline diagnostics.

In contrast, for longitudinal management of active glaucoma or post-operative uveitis, subtle IOP shifts of 2 mmHg determine whether to escalate or taper antiglaucoma medical therapy. Because the 90% confidence corridor exceeds 2.0 mmHg, switching between Tono-Pen and TonoVet across sequential rechecks introduces device-mediated artifacts that can obscure clinical progression or therapeutic response. Serial monitoring must use the same instrument model.

### 4.4. Sources of Heterogeneity and Methodological Considerations

Substantial statistical heterogeneity was observed across the single-arm cohorts (τ^2^ = 3.13–4.62, *I*_2_ > 90%). Our meta-regression confirmed that device modality was not the primary driver of the dispersion (*β* = −0.36 mmHg; *p* = 0.85), indicating that external methodological variables accounted for the between-study variance. Restraint methods ranged from halter restraint to chute systems [1,3,6,9,10]. Tight manual restraint around the neck or jugular groove compresses jugular venous return, directly elevating choroidal blood volume and IOP [1]. Furthermore, because camelid-specific calibration algorithms are lacking, studies relying on canine, equine, or human manufacturer modes might not be directly applicable to camelids [11]. However, insufficient reports have discussed IOP influenced by the single or combination of other factors, such as ambient temperature and wind exposure in field settings. Some authors have reported no differences in IOP by gender or age [1,2,3].

### 4.5. Strengths, Limitations, and Future Directions

A major methodological strength of this study is the implementation of a paired covariance sensitivity model combined with TOST equivalence testing. This approach resolved the common meta-analytic limitation of missing primary covariance metrics, avoiding the severe Type II error inflation that occurs when paired ocular data are treated as independent groups.

Several limitations remain. First, the lack of prospective protocol registration in a public repository (e.g., PROSPERO or OSF) is a methodological limitation that may limit transparency regarding potential post hoc analytical adjustments, although our analytical plan and eligibility criteria were defined a priori. Second, direct head-to-head paired datasets were restricted to two studies [3,10]. Thirdly, the primary literature did not report central corneal thickness (CCT), preventing quantitative meta-regression to control for corneal biomechanical confounding. Finally, manometric cannulation studies in camelids are lacking; therefore, the accuracy of the measurements relative to true intracameral hydrostatic pressure could not be determined.

Further investigations should provide experimental data to validate the protocol for using the two devices in various fields in alpacas. Reporting paired difference standard deviations (SD_diff_) and ultrasonic pachymetry data will further refine camelid-specific calibration equations and optimize ophthalmic care.

## 5. Conclusions

To our knowledge, this systematic meta-analysis provides the first basic image of intraocular pressure data in alpacas and llamas. The results indicate that the expected pooled IOP range in healthy alpacas is approximately 15.8 mmHg (95% CI: 14.0–18.0 mmHg), with no statistically significant difference between Tono-Pen and TonoVet readings, despite moderate heterogeneity. Further investigations into manometric calibration across varying pressure ranges are warranted to establish species-specific conversion algorithms and to optimize camelid ophthalmic care.

## Figures and Tables

**Table 1:** Summary of studies included in the meta-analysis of intraocular pressure (IOP) measured by Tono-Pen and TonoVet.

Study (Year/First Author)	Device	N (Eyes)	Mean IOP ± SD (mmHg)	Notes	References
Willis (2000)	Tono-Pen	36	14.85 ± 2.7	Applanation tonometry	[1]
Nuhsbaum (2000)	Tono-Pen	20	16.14 ± 3.74	Applanation tonometry	[6]
Webb (2006)	Tono-Pen	46	19.00 ± 4.00	Applanation tonometry	[9]
Pietro (2016)	Tono-Pen	46	15.30 ± 1.80	Applanation tonometry	[11]
McDonald (2017)	Tono-Pen	80	12.51 ± 2.78	Measured in healthy animals	[10]
McDonald (2017)	TonoVet	80	14.21 ± 2.73	Same study, rebound tonometry	[10]
Faulkner (2020)	TonoVet	92	17.20 ± 5.40	Different subgroups of healthy eyes	[3]
Faulkner (2020)	TonoVet	50	16.4 ± 5.3	Rebound tonometry, separate subgroup	[3]
Faulkner (2020)	Tono-Pen	50	17.6 ± 5.7	Applanation tonometry	[3]
–	Overall	–	–	Total = 9 datasets (6 Tono-Pen; 3 TonoVet)	

Abbreviations: SD = standard deviation; IOP = intraocular pressure. All measurements are reported as mean ± SD in mmHg. Data are extracted from published sources reporting clinically normal eyes without ocular pathology.

**Table 2:** Item-by-item JBI appraisal of included studies.

Study	Q1. Inclusion Criteria Clearly Defined?	Q2. Study Subjects and Setting Described in Detail?	Q3. Exposure Measured Validly and Reliably?	Q4. Objective, Standard Criteria Used for IOP Measurement?	Q5. Confounding Factors Identified?	Q6. Strategies to Deal with Confounding Stated?	Q7. Outcomes Measured Validly and Reliably?	Q8. Appropriate Statistical Analysis Used?	Overall Risk	Key Observations
Willis (2000) [1]	Y	Y	Y	Y	U	U	Y	Y	Low	Inclusion criteria stated; healthy adults only. Tono-Pen validated. No confounders (sedation, restraint) addressed. Statistics limited to descriptive.
Nuhsbaum (2000) [6]	Y	Y	Y	Y	U	Y	Y	Y	Low	Setting not fully described; no SD range reported. Instrument use described but training not mentioned; no confounding strategy.
Webb (2006) [9]	Y	Y	Y	Y	U	U	Y	Y	Low	Sample well described; Tono-Pen calibrated per protocol. Potential confounders (time of day, restraint) mentioned but not controlled.
Pietro (2016) [11]	Y	Y	Y	Y	Y	Y	Y	Y	Low	Most complete reporting. Confounders identified; strategies described but inconsistently applied. Both device types used.
McDonald (2017) [10]	Y	Y	Y	Y	Y	Y	Y	Y	Low	Inclusion criteria stated; healthy adults only. Tono-Pen validated. Confounders (sedation, restraint, and biomechanics) addressed; topical local anesthetic was used; general restraint by hand or muzzle.
Faulkner (2020) [3]	Y	Y	Y	Y	Y	Y	Y	Y	Low	Inclusion criteria stated; subgroup to control age; general restraint; sampling method may bring high variance; topical local anesthetic was used; Instrument use described but training not mentioned; No confounding strategy.

Y: yes; N: no; U: unclear. All six included studies achieve scores corresponding to a low risk of bias (scores ≥ 6/8). The four comparative trials meet all eight criteria (8/8), demonstrating clear inclusion criteria, detailed animal descriptions, validated instrumentation, explicit management of confounders, and sound statistical reporting [1,3,10,11]. The remaining two studies [6,9] are rated 7/8 and 6/8, respectively, primarily because they incompletely address specific confounders (such as restraint-induced stress or diurnal timing) and strategies to control them. No studies are deemed to have a high risk of bias.

**Table 3:** Subgroup meta-analysis and between-group difference in alpaca intraocular pressure measurements compiled by tonometry technique (Tono-Pen versus TonoVet).

Analysis Model/Comparison	Pooled Estimate (mmHg)	95% Confidence Interval	τ^2^	Q	df	*p*-Value
Part A: Subgroup Meta-Analysis (All eligible datasets, k = 9)						
Tono-Pen (k = 6, N = 278)	15.84	14.06 to 17.62	4.62	123.09	5	<0.001
TonoVet (k = 3, N = 222)	15.89	13.79 to 17.99	3.13	25.32	2	0.001
Between-group difference (TonoVet − Tono-Pen)	−0.36	−4.86 to 4.17	—	—	—	0.85
Part B: Head-to-Head Paired Comparison (Identical subjects, k = 2)						
Fixed-effect model (TonoVet − Tono-Pen)	+1.25	+0.48 to +2.02	—	5.95	1	0.015
Random-effects model (DerSimonian–Laird)	+0.58	−1.85 to +3.02	2.60	5.95	1	0.64

**Table 4:** Two one-sided tests (TOST) equivalence analysis across sensitivity corridors.

Study	Margin (±Δ)	*r* = 0.5 (*p*_TOST_)	*r* = 0.6 (*p*_TOST_)	*r* = 0.7 (*p*_TOST_)	*r* = 0.8 (*p*_TOST_)	Statistical Conclusion (*ɑ* = 0.05)
McDonald et al. (2017) [10]	±1.0 mmHg	0.987	0.993	0.998	>0.999	Not Equivalent
(N = 80, Diff: +1.70)	±1.5 mmHg	0.741	0.765	0.798	0.846	Not Equivalent
	±2.0 mmHg	0.167	0.140	0.106	0.064	Not Equivalent
	±2.5 mmHg	0.006	0.002	<0.001	<0.001	Equivalent (*r* ≥ 0.5)
Faulkner et al. (2020) [3]	±1.0 mmHg	0.601	0.612	0.629	0.656	Not Equivalent
(N = 50, Diff: +1.20)	±1.5 mmHg	0.351	0.335	0.311	0.274	Not Equivalent
	±2.0 mmHg	0.155	0.128	0.096	0.056	Not Equivalent
	±2.5 mmHg	0.051	0.034	0.018	0.006	Equivalent (r ≥ 0.6)

## Data Availability

The fully annotated Python code and R codes the aggregated secondary dataset supporting the findings of this meta-analysis are available upon reasonable request from the corresponding author.

## Abbreviations

The following abbreviations are used in this manuscript:

IOP	Intraocular pressure
SD	Standard deviation
CI	Confidence interval
CCT	Central corneal thickness
PRISMA	The Preferred Reporting Items for Systematic Reviews and Meta-Analyses
JBI	The Joanna Briggs Institute
MD	Mean difference
TOST	Two One-Sided Tests
